# Supplementary material for: Multiscale modeling of presynaptic dynamics from molecular to mesoscale
Source: PLoS Comput Biol. 2022 May 9;18(5):e1010068. doi: 10.1371/journal.pcbi.1010068 (PMC9119629; doi:10.1371/journal.pcbi.1010068)
Supplement: Attachment — Submitted filename: Garcia-Bartol-Sejnowski.docx [file pcbi.1010068.s002.docx]

Evaluation of the manuscript by J.W. Garcia, T.M. Bartol and T.J. Sejnowski entitled: Multiscale modeling of presynaptic dynamics from molecular to mesoscale

# Comments

This study is an attempt to develop a presynaptic model of processes determining the dynamics of transmission efficacy. First, the traces of free Ca2+ concentration were obtained that occur in response to various action potential stimulus trains from a Monte Carlo model of a hippocampal mossy fiber axon. Ca2+ was estimated as it diffuses from the voltage dependent Ca channel (VDCC) source, through the buffer, to the Ca2+ sensor in the SNARE complex and its time course determined at varying distance from a VDCC cluster with Ca2+ being buffered by calbindin. The calcium traces then drive deterministic state vector models of synaptotagmin 1 and 7 (Syt-1/7), which respectively mediate synchronous and asynchronous release in excitatory hippocampal synapses. This paper extends their earlier Monte Carlo studies of synaptic transmission, and it comes from a prominent group that worked on these and related problems for a long time. The methodology is well described and discussed. Moreover, there is a definitive need for simulations that combine Ca dynamics and release processes.

Authors stress the advantages of this approach over biological experiments in terms of both flexibility and precision when it comes to model validation are discussed. They also discuss disadvantages – a) Does Markov chain Monte Carlo framework accurately replicate the dynamics of diffusion and molecular interactions of these mechanisms? b) Is the molecular kinetics of the included species their true kinetics? and c) Are the molecular species and processes included the only relevant ones? That is needed and appropriate.

**Criticisms**

I have two basic issues with this paper:

First issue relates to Ca diffusion and how it is addressed. As authors state diffusion plays a key role in presynaptic processes. However, the question of how micro or nanostructures like cytoskeleton affect diffusion and ultimately release is just briefly mentioned. Why? Is it so time consuming to do some additional simulations with idealized geometry to answer the question how such intracellular micro-structures affect the diffusion and release? The importance of vesicles themselves (or of vesicular clusters) as diffusional barriers is not addressed at all. It should be. It is well documented that they play an important role in restricting diffusion. See work of Cooper et al., (1996) J Neurophysiol 75:2451; Glavinovic & Rabie, Pflügers Arch (2001) 443:132; Shahrezaei et al., (2006) J. Neurosci.26:13240. In central synapses individual vesicles play a less important role as diffusion barriers than in adrenal secretory cells, but they cluster. Doing additional simulations with vesicles included would be ideal, but even in the absence of new simulations it should be discussed, earlier work referred to and commented upon. Authors discuss local saturation of both the calbindin buffer and the PMCA pumps in the nanodomains near the VDCC cluster, where free Ca2+ reaches high levels and how it affects proximal Ca curve. Please expand on this in the context of slower diffusion caused by vesicular presence.

Second, this paper presents a model for hippocampal mossy fiber axon synapse with a given set of Ca binding-unbinding parameters. However, these parameters which determine the total binding ratio (the ratio of bound to free Ca2+) varies greatly in different secretory systems (Stuenkel (1994) J Physiol 481:251; Tank et al. (1995) J Neurosci 15:7940). Moreover, the binding ratio changes with cell development because the expression of Ca2+ buffers is developmentally regulated (Friauf (1993) J Comp Neurol 334:59; Lohman and Friauf (1996) J Comp Neurol 367:90. How would different Ca binding-unbinding parameters change Ca dynamics and release processes? Could authors comment on these issues, and if possible, do some simulations to answer them more completely. These changes would make this work significantly more interesting to a wider audience.
