# Supplementary material for: Multiscale modeling of presynaptic dynamics from molecular to mesoscale
Source: PLoS Comput Biol. 2022 May 9;18(5):e1010068. doi: 10.1371/journal.pcbi.1010068 (PMC9119629; doi:10.1371/journal.pcbi.1010068)
Supplement: Attachment — Submitted filename: Reviewer Feedback.docx [file pcbi.1010068.s003.docx]

Reviewer's Responses to Questions

Comments to the Authors:

Please note here if the review is uploaded as an attachment.

Reviewer #1: The study by Garcia et al. is an important contribution that makes it possible to scale-up the implication of synaptic designs to networks of neurons. They have chosen an important synapse, the mossy fiber synapse in the trisynaptic circuit of the hippocampus for their modeling investigation. There exists thousands of morphologically distinct synapses that play a diverse roles in information transfer across neurons. The suggests a strong causal link between synaptic morphology and function. Yet, there is often a disconnect between understanding at the level of synapses and neuronal circuit dynamics in both experimental studies and modeling studies. On the other hand it is unrealistic to simulate in great spatial and physiological detail activity at the synapses when computing activity of hundreds of neurons. This manuscript attempts to do fill this gap.

I think, over-all results of the phenomenological are a good quantitative fit to detailed spatiotemporal calculations and therefore useful for scaling up the numbers of synapses in simulations and computation with neuronal networks. I do have a few concerns regarding the specifics of the model that are listed below.

• I see that various train stimuli have been explored, however it be useful to show comparison of the phenomeological model with realistic model for more realistic trains of activity (variable frequency and noisy stimulus). Since the parameters of the phenomenological models are retro-fit to specific data, how do the authors envision incorporating it in a network (since thats the point of this study) which sees time varying stimulus?

The ramp-probe spike protocol was our way of extracting as much information about facilitation as possible from short spike trains by measuring both the rate of accumulation of facilitation for different frequencies of spike ramps and how quickly facilitation decays to baseline for different delays of probe spikes. We expect that facilitation in response to random spike trains of similar lengths would follow our model’s predictions since such spike trains would fall within the space spanned by our ramp-spike protocol. However, behavior of the detailed MCell model may start to diverge from our phenomenological model for longer trains, which fall outside of the space we tested. Running longer MCell simulations to better characterize steady-state behavior of the facilitation function is a priority in the next stage of this project. Testing with long, randomized spike trains should also be a priority. We added some of this to the Discussion section.

• Figure 9. Description of this figure in the results and and figure both are hard to understand and need to be expanded.

The figure description has been expanded to emphasize the existence of multiple traces in each panel and the difference between spike ramps (dark colors) and probe spikes (light colors):

Fig 9. Empirical Facilitation in Synchronous and Asynchronous Release Rates.

Release rate profiles facilitate in response to single spikes (A) or to spike ramps (B,C). Probe spikes of increasing ISI reveal how facilitation then decays back toward baseline after a delay. Spike events themselves not shown. Synchronous release rate profiles shown in blue. Asynchronous profiles shown in red. Profiles from multiple runs are overlaid in each panel. Dark colors represent response to initial spike or spike ramp (common to all traces on a plot). Light colors represent profiles from different runs in response to single probe spikes at different ISIs following the initial spike or ramp. (A) After single spike, paired-pulse facilitation decays with increasing ISI (probe ISIs of 2, 5, 10, 20, 50, 100, 200 ms). (B) 5-spike ramp with a 5-ms ISI shows strong facilitation in release rate (dark colors) followed by rapid decay seen at the probe spikes (light colors). (C) 5-spike ramp with a 20-ms ISI shows weaker facilitation in the ramp phase but a similar rate of decay at the probe spikes. Note the orders of magnitude difference in scale between synchronous and asynchronous release rates, as well as the change in scale from (A) to (B,C).

• I did not see a description of the mossy fiber synapse design at all. This is a surprising omission given that the model is of that of MF. For that matter, how is the model different from a schaffer collateral? What specifics of the MF have been included (RRP size, number of release sites etc.) MFs show distinct short-term plasticity profiles as described in detail by several papers from Peter Jonas’s lab, Katalin Toth lab and Joachim Lu?bke lab. This needs to be included

The synapse studied in our paper, on which the MCell model was based, is from the Schaffer collateral, not the mossy fiber. An incorrect reference to the mossy fiber axon has been fixed in the abstract of the manuscript. The structure of the synapse in the MCell model used in this study is described in the paper, with a visualization of the vesicle layout in Fig 2A. We apologize for the confusion.

Minor comments

• References list seems really long, is a more concise list possible?

We were able to trim out some references that we found redundant.

• It will be useful if the model is available on a public repository like a modelDB. Also final parameters (taus of decay and facilitation etc.) of the model that fit the detailed MCell simulations for various stimuli could be presented in tabular format for ease of use.

We plan to upload the MCell model to an online repository (<http://mcell.cnl.salk.edu/models/presynaptic-dynamics-2022-1/>) upon acceptance of this paper.

The final parameters are available in Tables 2 and 3 for the 400-nm cluster of 100 VDCCs. Parameter values for other distances are plotted in S7 Fig and can be found in the MATLAB data available on GitHub (<https://github.com/soiens24/Presynaptic_Release_Rates>).

Both URLs are now listed in the Data and Code Availability statement:

All data files and code for generating all figures in the manuscript and Supporting Information have been uploaded to a publicly accessible repository on GitHub at the following URL: <https://github.com/soiens24/Presynaptic_Release_Rates>. The MCell/CellBlender project file for the MCell model of the Schaffer collateral axon used in this paper is available at the MCell model repository at the following URL: <http://mcell.cnl.salk.edu/models/presynaptic-dynamics-2022-1/>.

Reviewer #2: Evaluation of the manuscript by J.W. Garcia, T.M. Bartol and T.J. Sejnowski entitled: Multiscale modeling of presynaptic dynamics from molecular to mesoscale

Comments

This study is an attempt to develop a presynaptic model of processes determining the dynamics of transmission efficacy. First, the traces of free Ca2+ concentration were obtained that occur in response to various action potential stimulus trains from a Monte Carlo model of a hippocampal mossy fiber axon. Ca2+ was estimated as it diffuses from the voltage dependent Ca channel (VDCC) source, through the buffer, to the Ca2+ sensor in the SNARE complex and its time course determined at varying distance from a VDCC cluster with Ca2+ being buffered by calbindin. The calcium traces then drive deterministic state vector models of synaptotagmin 1 and 7 (Syt-1/7), which respectively mediate synchronous and asynchronous release in excitatory hippocampal synapses. This paper extends their earlier Monte Carlo studies of synaptic transmission, and it comes from a prominent group that worked on these and related problems for a long time. The methodology is well described and discussed. Moreover, there is a definitive need for simulations that combine Ca dynamics and release processes.

Authors stress the advantages of this approach over biological experiments in terms of both flexibility and precision when it comes to model validation are discussed. They also discuss disadvantages – a) Does Markov chain Monte Carlo framework accurately replicate the dynamics of diffusion and molecular interactions of these mechanisms? b) Is the molecular kinetics of the included species their true kinetics? and c) Are the molecular species and processes included the only relevant ones? That is needed and appropriate.

Criticisms

I have two basic issues with this paper:

First issue relates to Ca diffusion and how it is addressed. As authors state diffusion plays a key role in presynaptic processes. However, the question of how micro or nanostructures like cytoskeleton affect diffusion and ultimately release is just briefly mentioned. Why? Is it so time consuming to do some additional simulations with idealized geometry to answer the question how such intracellular micro-structures affect the diffusion and release? The importance of vesicles themselves (or of vesicular clusters) as diffusional barriers is not addressed at all. It should be. It is well documented that they play an important role in restricting diffusion. See work of Cooper et al., (1996) J Neurophysiol 75:2451; Glavinovic & Rabie, Pflügers Arch (2001) 443:132; Shahrezaei et al., (2006) J. Neurosci.26:13240. In central synapses individual vesicles play a less important role as diffusion barriers than in adrenal secretory cells, but they cluster. Doing additional simulations with vesicles included would be ideal, but even in the absence of new simulations it should be discussed, earlier work referred to and commented upon. Authors discuss local saturation of both the calbindin buffer and the PMCA pumps in the nanodomains near the VDCC cluster, where free Ca2+ reaches high levels and how it affects proximal Ca curve. Please expand on this in the context of slower diffusion caused by vesicular presence.

We did include synaptic vesicles capable of obstructing the buffered diffusion of calcium, as shown in Fig 2A, and we verified that the effective rate of diffusion through the presynaptic space matches what is observed experimentally. A paragraph was added to the paper a few paragraphs after Fig 2 that emphasizes our considerations around the effects of vesicles/cytoskeleton/buffer on the diffusion of calcium ions. Some of the references you suggested have been included.

Second, this paper presents a model for hippocampal mossy fiber axon synapse with a given set of Ca binding-unbinding parameters. However, these parameters which determine the total binding ratio (the ratio of bound to free Ca2+) varies greatly in different secretory systems (Stuenkel (1994) J Physiol 481:251; Tank et al. (1995) J Neurosci 15:7940). Moreover, the binding ratio changes with cell development because the expression of Ca2+ buffers is developmentally regulated (Friauf (1993) J Comp Neurol 334:59; Lohman and Friauf (1996) J Comp Neurol 367:90. How would different Ca binding-unbinding parameters change Ca dynamics and release processes? Could authors comment on these issues, and if possible, do some simulations to answer them more completely. These changes would make this work significantly more interesting to a wider audience.

Our model is of a Schaffer collateral axon, not a mossy fiber, but we did find and correct a mention of the mossy fiber in the abstract. We apologize for the confusion. As far as changing the kinetics and expression levels of calbindin, we expect that it might affect the magnitude and time constants of the exponential components of the release rate profiles (and maybe the number of components), but these new values could be easily determined using the same methods discussed in the paper and wouldn’t affect the phenomenological formula for release rate profiles qualitatively. We added a mention of this issue to the Discussion.
